# ACSM5 inhibits ligamentum flavum hypertrophy by regulating lipid accumulation mediated by FABP4/PPAR signaling pathway

**DOI:** 10.1186/s13062-023-00436-z

**Published:** 2023-11-14

**Authors:** Yanlin Cao, Jianjun Li, Sujun Qiu, Songjia Ni, Yang Duan

**Affiliations:** 1 Department of Spine Surgery, Zhujiang Hospital, Southern Medical University, Guangzhou, China; 2 Department of Orthopaedic Trauma, Zhujiang Hospital, Southern Medical University, Guangzhou, China

**Keywords:** Ligamentum flavum hypertrophy, ACSM5, FABP4, Lipid accumulation

## Abstract

**Background:**

Ligamentum flavum (LF) hypertrophy is the main cause of lumbar spinal canal stenosis (LSCS). Previous studies have shown that LF hypertrophy tissue exhibits abnormal lipid accumulation, but the regulatory mechanism remains unclear. The objective of this study was to explore the function and potential mechanism of ACSM5 in LF lipid accumulation.

**Methods:**

To assess the ACSM5 expression levels, lipid accumulation and triglyceride (TG) level in LF hypertrophy and normal tissue, we utilized RT-qPCR, western blot, oil red O staining, and TG assay kit. The pearson correlation coefficient assay was used to analyze the correlation between ACSM5 levels and lipid accumulation or TG levels in LF hypertrophy tissue. The role of ACSM5 in free fatty acids (FFA)-induced lipid accumulation in LF cells was assessed in vitro, and the role of ACSM5 in LF hypertrophy in mice was verified in vivo. To investigate the underlying mechanisms of ACSM5 regulating lipid accumulation in LF, we conducted the mRNA sequencing, bioinformatics analysis, and rescue experiments.

**Results:**

In this study, we found that ACSM5, which was significantly down-regulated in LF tissues, correlated with lipid accumulation. In vitro cell experiments demonstrated that overexpression of ACSM5 significantly inhibited FFA-induced lipid accumulation and fibrosis in LF cells. In vivo animal experiments further confirmed that overexpression of ACSM5 inhibited LF thickening, lipid accumulation, and fibrosis. Mechanistically, ACSM5 inhibited lipid accumulation of LF cells by inhibiting FABP4-mediated PPARγ signaling pathway, thereby improving hypertrophy and fibrosis of LF.

**Conclusions:**

our findings elucidated the important role of ACSM5 in the regulation of LF lipid accumulation and provide insight into potential therapeutic interventions for the treatment of LF hypertrophy. This study further suggested that therapeutic strategies targeting lipid deposition may be an effective potential approach to treat LF hypertrophy-induced LSCS.

**Supplementary Information:**

The online version contains supplementary material available at 10.1186/s13062-023-00436-z.

## Introduction

Lumbar spinal canal stenosis (LSCS) is a degenerative spinal disease that commonly affects elderly patients, causing clinical symptoms such as low back pain, radiation leg pain, and intermittent claudication [1]. However, most clinical symptoms arise due to compression of the spinal cord, nerve roots, or cauda equina. A spinal ligament called the ligamentum flavum (LF) covers the posterior and lateral parts of the dural sac, and its abnormal hypertrophy is a key factor leading to nerve compression and one of the primary causes of symptoms in patients with LSCS [2, 3]. Despite numerous studies exploring the mechanism of LF hypertrophy, the exact molecular regulation remains unclear.

Recent studies have revealed that abnormal lipid metabolism is a crucial cause of the occurrence and development of various diseases, such as cancer [4], non-alcoholic fatty liver disease [5], cardiovascular and cerebrovascular diseases [6]. High performance-liquid chromatography and mass spectrometry have demonstrated that total lipid accumulation and TG in LF hypertrophy tissue are significantly increased and significantly positively correlated with the thickness of LF hypertrophy tissue in patients with LSCS, and the removal of accumulated lipids in the LF hypertrophy tissue would be a better alternative to laminectomy surgical treatment [7]. Moreover, the relationship between obesity and LF hypertrophy is well established [8], but little is known about the mechanistic factors that control excess lipid accumulation in LF cells. Thus, understanding the mechanism of lipid accumulation in the LF hypertrophy tissue and searching for effective intervention targets for lipid accumulation may provide effective intervention measures other than surgical treatment for LSCS.

Acyl-CoA synthetase medium chain family member 5 (ACSM5) is located in the mitochondrial matrix and participates in the first step of fatty acid metabolism [9]. However, physiological and pathological properties of ACSM5 are still largely ignored in the past. Recently, the role of ACSM5 in human diseases has gradually attracted attention, and it is found to be involved in the occurrence and development of various diseases, such as breast cancer [10], attention-deficit/hyperactivity disorder [11], and myocardial infarction [12]. Our previous study showed that overexpression of ACSM5 significantly inhibited the proliferation, anti-apoptosis, and fibrosis in LF cells, indicating that ACSM5 played protective roles in LF hypertrophy [13]. However, it remains unclear how ACSM5 affects lipid accumulation of LF. Our study aimed to examine the function and underlying mechanism of ACSM5 in lipid accumulation of LF, and to provide a potentially effective non-surgical treatment strategy for LSCS caused by LF hypertrophy.

## Materials and methods

### Human LF samples

This study was authorized by the Institutional Research Ethics Committee of the Zhujiang Hospital of Southern Medical University (Approval No.: 2021-ky-122-01). Informed consent was obtained from all patients who participated in the study. A total of 29 LF tissues including LF hypertrophy and normal tissues were collected from patients undergoing lumbar spine surgery at Zhujiang Hospital of Southern Medical University (Guangzhou, China). The LF hypertrophy tissues (๥4 mm thickness) were collected from LSCS patient with LF hypertrophy, and LF normal tissues (≤ 4 mm thickness) were collected from age- and gender-matched lumbar disc herniation (LDH) patient without LF hypertrophy as control. All patients who were enrolled in the study did not have any underlying diseases such as cancer, heart disease, kidney disease, rheumatism and autoimmune diseases. Before surgery, all patients underwent magnetic resonance imaging scan to confirm the thickness of the LF, and all LF samples were obtained from the anatomical region (L4/5). Lipid accumulation and TG levels in LF were assessed using oil red O staining and TG assay kit, respectively.

### Oil red O staining

The lipid content within intra-tissue and intracellular of the LF was determined using the Improved oil red O staining kit (C0158M, Beyotime, Shanghai, China) according to the manufacturer’s recommended protocol. In briefly, the treated cells were collected and fixed with 4% paraformaldehyde (P0099, Beyotime, Shanghai, China) for 10 min. Meanwhile, fresh LF tissue was embedded in liquid nitrogen with OCT embedding medium (Sigma-Aldrich, Germany) after dehydration with sucrose gradient, and then sectioned using a freezing microtome (HS-LD5720-B Type, Shenyang Hengsong Technology Co. LTD, Shenyang, China). The fixed cells and the sections were then stained with the oil Red O staining working solution for 20 min. Subsequently, nuclei were counterstained with hematoxylin staining solution (C0107, Beyotime, Shanghai, China) after washing with staining wash solution and PBS, respectively. The stained sections or cells were imaged under Microscope Imaging Software (Leica). Finally, the images were converted to an RGB stack in ImageJ software (NIH, United States), and then the threshold was adjusted so that the red droplets turned into the black dot against a white background, thus measuring the red lipid droplets area.

### Triglyceride (TG) assays

The intra-tissue and intracellular TG content of the LF was measured using the triglyceride assay kit (BC0625, Solarbio, Beijing, China). In brief, LF tissues or cells were homogenized or sonicated, centrifuged at 8000 g for 10 min at 4℃, and the resulting supernatant was used for the assay with the recommended detection reagents according to the manufacturer’s recommended protocol. Finally, absorbance values were measured using the infinite® F50 microplate reader (Tecan) with a wavelength of 420 nm. Data were normalized by tissue weight or cell number appropriate. All experiments were performed in at least three biological replicates.

### Real-time quantitative PCR (RT-qPCR)

Total RNA was extracted from the LF cells using Trizol reagents (Invitrogen, Carlsbad, CA), and then reverse transcribed into cDNA using the PrimeScript™ RT reagent Kit with gDNA Eraser (Takara, Dalian, China). RT-qPCR amplification was performed on a LightCycler® 96 RT-qPCR instrument (Roche) using SYBR Green qPCR Mix (Beyotime, Shanghai, China). The β-actin was used as an internal control, and the relative mRNA expression levels were evaluated by 2^−ΔΔCT^ method. The primer sequences used in RT-qPCR were as follows in Table 1. All experiments were performed in at least three biological replicates.

**Table 1:** The primers sequencing was used in RT-qPCR

Name	Forward primer	Reverse primer
β-actin	CTACCTCATGAAGATCCTCACCGA	TTCTCCTTAATGTCACGCACGATT
ACSM5	ACACTGGCTGGGTGAAGG	ACAGCAGAGGGTGGTTATCG
FABP4	ACTGGGCCAGGAATTTGACG	CTCGTGGAAGTGACGCCTT
PPARγ	CAGCACCACCGATCAGAAGA	TCCCATTTCCGAGGAGGGAT
ACC	ATGTCTGGCTTGCACCTAGTA	CCCCAAAGCGAGTAACAAATTCT
FASN	GGAGGTGGTGATAGCCGGTAT	TGGGTAATCCATAGAGCCCAG

### Western blot

Western blotting was conducted as previous described (Cao, Zhan et al. 2021). Briefly, total protein wad extracted using precooled RIPA lysis buffer containing protease inhibitors (Beyotime, Shanghai, China). Equal volumes of proteins were separated by SDS-PAGE gel electrophoresis and then transferred to polyvinylidene fluoride (PVDF) membranes (Millipore, USA). The membranes were then blocked with 2% BSA buffer for 1 h at room temperature, incubated with appropriate primary antibody overnight at 4 °C. After washing the membranes three times, incubated with diluted conjugated secondary antibody for 1 h at room temperature. Finally, membranes were imaged and analyzed by a gel imaging system (Bio-Rad) with ECL Kit (Pierce, Thermo Fisher Scientific, IL, USA) and ImageJ software (NIH, United States). Primary antibodies used in the study were ACSM5 (1:200, FNab00112, Wuhan Fine Biotech Co., Ltd), FABP4 (1:1000, ab92501, Abcam), FASN (1:500, ab128870, Abcam), ACC (1:1000, ab45174, Abcam), collagen I (1:1000, ab260043, Abcam), collagen III (1:1000, ab7778, Abcam), α-SMA (1:2000, ab124964, Abcam), PPARγ (1:500, ab45036, Abcam), and β-actin (1 µg/ml, ab8226, Abcam). All experiments were performed in at least three biological replicates.

### Adenovirus construction and Infection

The ACSM5 adenovirus (ADV-ACSM5), FABP4 adenovirus (ADV-FABP4) and their negative control adenovirus (ADV-NC) were constructed and packaged by Ribobio Inc. (Guangzhou, Guangdong, China). LF cells were isolated and cultured from the normal LF tissues of patients with LDH according to our previously described method (Cao, Zhan et al. 2021). LF cells were infected with the adenovirus according the manufacturer’s instructions, and the infection efficiency was affirmed by RT-qPCR or western blot.

### Cell counting Kit-8 assay

Cell proliferation ability was detected by Enhanced Cell Counting Kit-8 (Beyotime, Shanghai, China) according to the manufacturer’s recommended protocol. In brief, the treated cells were incubated with the enhanced cell counting kit-8 solution for 1 h, and then the absorbance value was measured using the infinite® F50 microplate reader (Tecan) at the wavelength of 450 nm. All experiments were performed in at least three biological replicates.

### Transcriptomic RNA sequencing and data analysis

Total RNAs were extracted from LF cells of ADV-NC + FFA group and ADV-ACSM5 + FFA group using TRIzol reagent (Invitrogen) for transcriptomic RNA sequencing and analysis (Baimake Biotechnology Co., Ltd.). Library sequencing was performed on the Illumina HiSeq Xten platform (Illumina, San Diego, California, USA). The differentially expressed genes (DEGs) between groups were performed using EBseq R package, and Kyoto Encylopaedia of Genes and Genomes (KEGG) pathway enrichment analysis of DEGs was conducted using DAVID (Database for Annotation, Visualization and Integrated Discovery) [14]. The screening criteria for DEG were fold changes (FCs) ≥ 1.2 or ≤-1.2 and false discovery rates (FDRs) < 0.05.

### Animal experiments

All animal experimental protocols were approved by the Ethics Committee for Animal Research of the Zhujiang Hospital of Southern Medical University. C57BL/6 male mice (8-week-old, SPF) were purchased from the Experimental Animal Center of Southern Medical University. A mouse model of LF hypertrophy was constructed according to our previously reported method that utilizes the hydrophobia property of mice [15, 16]. Negative control (NC) mice were kept in the same environment as the mice used to create the LF hypertrophy model, except that there was no water at the bottom of the container [15, 16]. To determine the molecular mechanism by which ACSM5 affects ligamentous hypertrophy in mice by regulating FABP4, we randomly divided the LF hypertrophy model mice into model group, model + adeno-associated virus (AAV)-ACSM5 group, and model + AAV-ACSM5 + AAV-FABP4 group. After seven weeks of modeling, a longitudinal skin incision was made on the lumbar spine of anesthetized mice, and the dorsal paravertebral muscles were excised from the spinous process and lamina, exposing the LF at L5/6 under an operating microscope. Subsequently, AAV-NC, AAV-ACSM5 or AAV-FBAP4 (1 × 10^12^ vg/ml, 3 µl) were injected into the L5/6 LF of the corresponding mice using a microinjector (NF36BV 36GA, NanoFil, United States) according to the grouping need. The AAV-NC, AAV-ACSM5 and AAV-FBAP4 were obtained from Hanbio Biotechnology (Shanghai, China). After 4 weeks of AAV injection, the mice were euthanized, and the LF tissues of L5/6 vertebral bodies were collected for hematoxylin and eosin (H&E) staining and molecular biological analysis.

### H&E staining

H&E staining was performed to measure the area of the LF. Briefly, the LF tissues from the mice were removed of excess vegetation, fixed with 4% paraformaldehyde (Beyotime, shanghai, China) overnight, and embedded in paraffin and sliced. After sectioning, H&E staining was performed according to the protocol of the H&E kit (Beyotime, shanghai, China). Subsequently, the images were observed and captured under a microscope (Leica). Finally, quantitative analyses of the LF areas were obtained using ImageJ software (NIH, United States). Each sample was detected three times and its average value was taken.

### Statistical analysis

All data were presented as the means ± standard deviation (SD). All the statistical analyses were performed using the GraphPad Prism 6.0 (GraphPad Software, La Jolla, USA). Statistical significance between groups were analyzed using Student’s t-test or one-way ANOVA. The p-values < 0.05 was considered as significant difference.

## Results

### Low expression of ACSM5 in LF hypertrophy tissue was significantly correlated with lipid accumulation

**Fig. 1:**
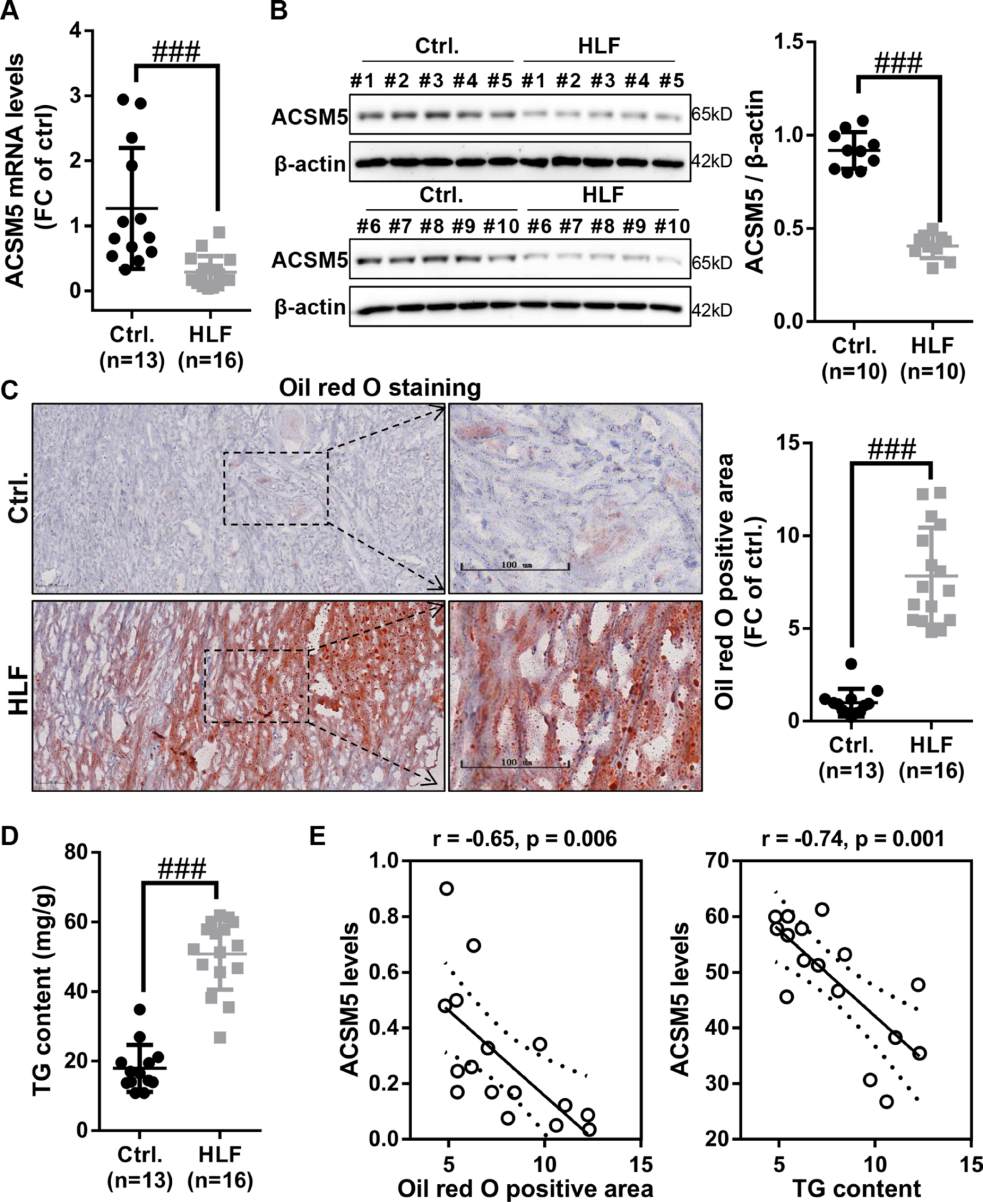
The downregulation of ACSM5 in LF hypertrophy tissues was significantly correlated with lipid accumulation. (**A**) RT-qPCR was used to detect the mRNA expression levels of ACSM5 in LF hypertrophy tissue (n = 16) and normal tissue (n = 13). (**B**) Western blot was used to detect the mRNA expression level of ACSM5 in LF hypertrophy tissue (n = 10) and normal tissue (n = 10). (**C**) Lipid accumulation in the LF hypertrophy tissue (n = 16) and normal tissue (n = 13) was detected by oil red O staining. Bar size = 100 nm. (**D**) TG levels in the LF hypertrophy tissue (n = 16) and normal tissue (n = 13) was detected using TG assay kit. (**E**) Pearson Correlation Coefficient Analysis showed that the expression levels of ACSM5 in LF hypertrophy tissues was negatively correlated with TG levels and lipid accumulation. Ctrl., ligamentum flavum normal tissues. HLF, hypertrophy tissues of ligamentum flavum. TG, triglyceride. ^###^P < 0.001

Previous studies have shown that abnormal lipid accumulation is closely related to LF hypertrophy in patients with intervertebral disc stenosis, and understanding the mechanism of abnormal lipid accumulation in the LF may provide intervention measures other than surgery for patients with disc stenosis caused by LF hypertrophy [7]. To investigate whether ACSM5 played a role in abnormal lipid accumulation of the LF, the mRNA and protein expression levels of ACSM5 in LF hypertrophy and normal tissues was examined by RT-qPCR and western blot. The results demonstrated that the mRNA and protein expression levels of ACSM5 were lower in LF hypertrophy tissues than in LF normal tissues (Fig. 1A and B). Lipid accumulation of LF tissues was evaluated using oil red O staining and TG assay kit. The results revealed that the oil red O positive area and TG levels in LF hypertrophy tissues were significantly higher than those in the LF normal tissues (Fig. 1C and D), suggesting abnormal lipid accumulation occurs in LF hypertrophy tissue. Furthermore, Pearson correlation coefficient analysis showed that the expression levels of ACSM5 were negatively correlated with oil red O positive area and TG levels in the LF hypertrophy tissues (Fig. 1E). Taken together, our data demonstrated that low expression of ACSM5 might be involved in lipid accumulation of LF hypertrophy tissue.

### Overexpression of ACSM5 inhibited FFA-induced lipid accumulation and fibrosis in LF cells

**Fig. 2:**
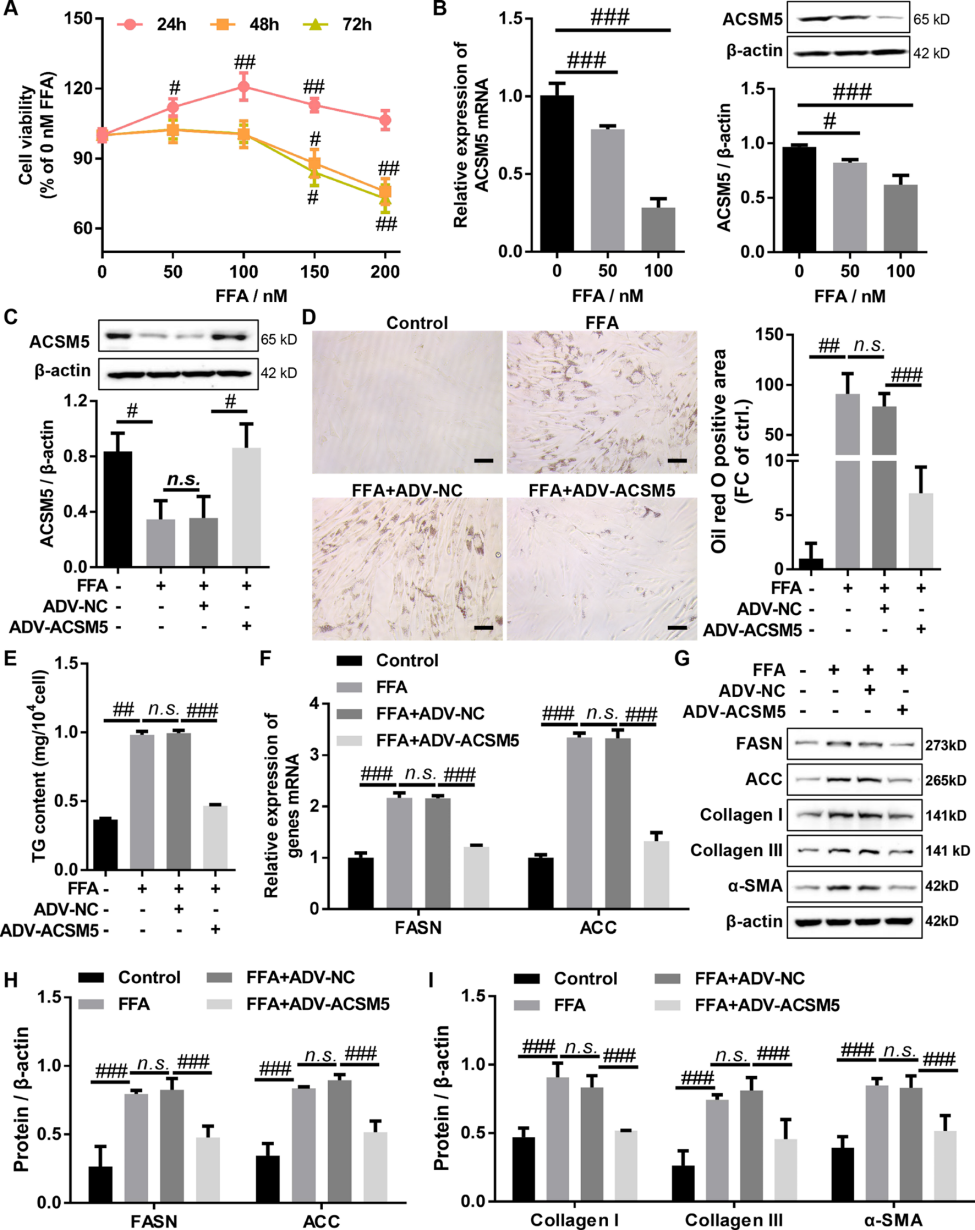
Overexpression of ACSM5 inhibited FFA-induced lipid accumulation and fibrosis in LF cells. (**A**) CCK-8 assay was used to detect the effect of FFA on the proliferation of LF cells. (**B**) LF cells were treated with different concentrations of FFA for 48 h, and the mRNA and protein expression levels of ACSM5 in LF cells were detected by RT-qPCR and western blot. (**C**) ADV-ACSM5- or ADV-NC-infected LF cells were treated with FFA for 48 h, and the protein expression levels of ACSM5 in LF cells were detected by western blot. (**D**) ADV-ACSM5- or ADV-NC-infected LF cells were treated with FFA for 48 h, and oil red O staining was used to detect intracellular lipid accumulation in LF cells. Bar size = 100 nm. (**E**) ADV-ACSM5- or ADV-NC-infected LF cells were treated with FFA for 48 h, and TG assay kit was used to detect TG levels in LF cells. (**F**) ADV-ACSM5- or ADV-NC-infected LF cells were treated with FFA for 48 h, and the mRNA expression levels of FASN and ACC in LF cells were detected by RT-qPCR. (**G** and **H**) ADV-ACSM5- or ADV-NC-infected LF cells were treated with FFA for 48 h, and the protein expression of FASN and ACC in LF cells was detected by western blot. (**G** and **I**) ADV-ACSM5- or ADV-NC-infected LF cells were treated with FFA for 48 h, and the protein expression of collagen I, collagen III and α-SMA in LF cells was detected by western blot. LF, ligamentum flavum. FFA, free fatty acids. ADV, adenovirus. NC, negative control. ^n.s^.P > 0.05, ^#^P < 0.05, ^##^P < 0.01, and ^###^P < 0.001

To explore the role of ACSM5 in regulating lipid accumulation in LF, LF cells were treated with different concentrations of FFA. CCK-8 results showed that the proliferation of LF cells was significantly increased after treatment with 50, 100 and 150 nM FFA for 24 h (Fig. 2A). And treatment with 50 and 100 nM FFA for 48 and 72 h did not significantly change the proliferation of the LF cells, while treatment with 150 and 200 nM FFA for 48 and 72 h significantly inhibited the proliferation of the LF cells (Fig. 2A). In addition, the results of RT-qPCR and western blot demonstrated that the mRNA and protein expression levels of ACSM5 were significantly reduced in LF cells treated with 50 nM and 100 nM FFA for 48 h, which decreased with increasing concentration (Fig. 2B). Therefore, LF cells were treated with 100nM FFA for 48 h to carry out subsequent experiments. Subsequently, the LF cells infected with ADV-NC or ADV-ACSM5 were treated with 100 nM FFA for 48 h, the protein expression of ACSM5 was detected by western blot. The results indicated that ADV-NC had no significant effect on FFA-induced downregulation of ACSM5 protein in LF cells, but ADV-ACSM5 could reverse the inhibitory effect of FFA on ACSM5 protein levels (Fig. 2C). Oil red O staining and TG assay kit were used to evaluate the effect of ACSM5 on lipid accumulation in LF cells, and found that FFA induced lipid accumulation in LF cells, this induction effect was significantly reversed by ACSM5 overexpression (Fig. 2D and E). At the same time, the results of RT-qPCR and western blot demonstrated that FAA significantly increased the mRNA and protein expression levels of lipid synthesis genes (FASN and ACC) in LF cells, this promotion effect was significantly reversed by overexpression of ACSM5 (Fig. 2F, G and H). In addition, western blot was used to detect the protein expression of fibrosis-related genes (Collagen I, Collagen III, and α-SMA). The results indicated that FAA could significantly increase the protein expression levels of Collagen I, Collagen III and α-SMA in the LF cells, this promotion effect could be hindered by overexpression of ACSM5 (Fig. 2H and I). Overall, the above results demonstrated that overexpression of ACSM5 significantly suppressed FFA-induced lipid accumulation and fibrosis in LF cells.

### Overexpression of ACSM5 inhibited FABP4/PPAR signaling in FFA-induced LF cells

**Fig. 3:**
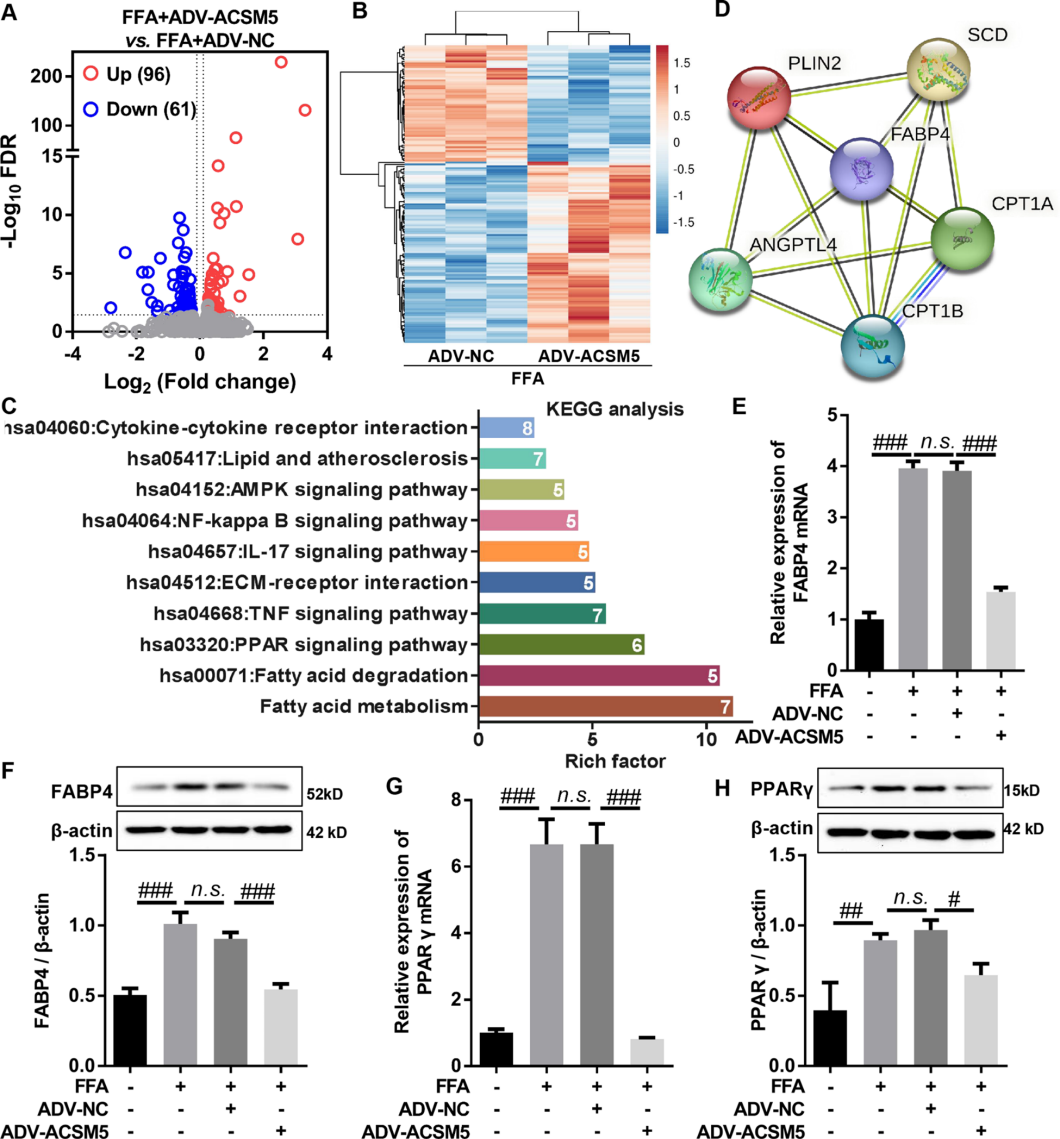
Overexpression of ACSM5 inhibited FABP4/PPAR signaling pathway in FFA-induced LF cells. (**A**) Volcano plot showed the mRNAs expression profile between FFA + ADV-NC group and FFA + ADV-ACSM5 group in LF cells. |FC| > 1.2 and FDR < 0.05 were used to screen differential expressed mRNAs. (**B**) The cluster heatmap showed the differential mRNA expression profiles between FFA + ADV-NC group and FFA + ADV-ACSM5 group in LF cells. (**C**) KEGG enrichment analysis was performed on the differential expressed genes between FFA + ADV-NC group and FFA + ADV-ACSM5 group in LF cells. (**D**) The differential expressed genes enriched in PPARγ signaling pathway were analyzed by PPI network. (**E**) ADV-ACSM5- or ADV-NC-infected LF cells were treated with FFA for 48 h, and the mRNA expression of FABP4 in LF cells was detected by RT-qPCR. (**F**) ADV-ACSM5- or ADV-NC-infected LF cells were treated with FFA for 48 h, and the protein expression levels of FABP4 in LF cells were detected by western blot. (**G**) ADV-ACSM5- or ADV-NC-infected LF cells were treated with FFA for 48 h, and the mRNA expression levels of PPARγ in LF cells were detected by RT-qPCR. (**H**) ADV-ACSM5- or ADV-NC-infected LF cells were treated with FFA for 48 h, and the protein expression levels of PPARγ in LF cells were detected by western blot. LF, ligamentum flavum. FFA, free fatty acids. ADV, adenovirus. NC, negative control. ^n.s^.P > 0.05, ^#^P < 0.05, ^##^P < 0.01, and ^###^P < 0.001

To explore the potential molecular mechanism of ACSM5 inhibiting lipid accumulation in LF cells, we conducted transcriptome sequencing analysis on ADV-NC or ADV-ACSM5-infected LF cells treated with 100nM FFA for 48 h, and FC > 1.2 & FDR < 0.05 were used as screening criteria for DEGs between groups. Compared with ADV-NC + FFA group, there were 157 DEGs in ADV-ACSM5 + FFA group, of which 96 were significantly up-regulated and 61 were significantly down-regulated (Fig. 3A and B). We performed KEGG analysis for those DEGs, and found that they were mainly enriched in signaling pathways related to lipid metabolism and inflammation, such as Fatty acid metabolism, Fatty acid degradation, PPAR signaling pathway, TNF signaling pathway, ECM-receptor interaction, IL-17 signaling pathway, NF-kappa B signaling pathway, AMPK signaling pathway, Lipid and atherosclerosis, and Cytokine-cytokine receptor interaction (Fig. 3C). Previous studies have shown that PPAR signaling pathway plays key roles in lipid accumulation and fibrosis diseases [17–19]. Our KEGG enrichment analysis showed that 6 DEGs (FABP4, CPT1A, CPT1B, ANGPTL4, PLIN2 and SCD) were enriched in PPAR signaling pathway and interacted with each other (Fig. 3D). Furthermore, PPAR pathway information generated by KEGG showed that FABP4 was an upstream gene of PPAR signaling pathway (Fig. S1). Meanwhile, numerous studies have shown that FABP4 positively regulates PPARγ signaling [20–22], which plays an important role in lipid metabolism [23, 24]. RT-qPCR and western blot results showed that FFA significantly increased the mRNA and protein expression of FABP4 and PPARγ in LF cells, this promotion effect could be significantly reversed by overexpression of ACSM5 (Fig. 3E, F, G and H). Taken together, our data demonstrated that overexpression of ACSM5 inhibited FABP4/PPARγ signaling pathway in FFA-induced LF cells.

### Overexpression of ACSM5 inhibited FFA-induced lipid accumulation and fibrosis by regulating FABP4-mediated PPAR signaling in LF cells

**Fig. 4:**
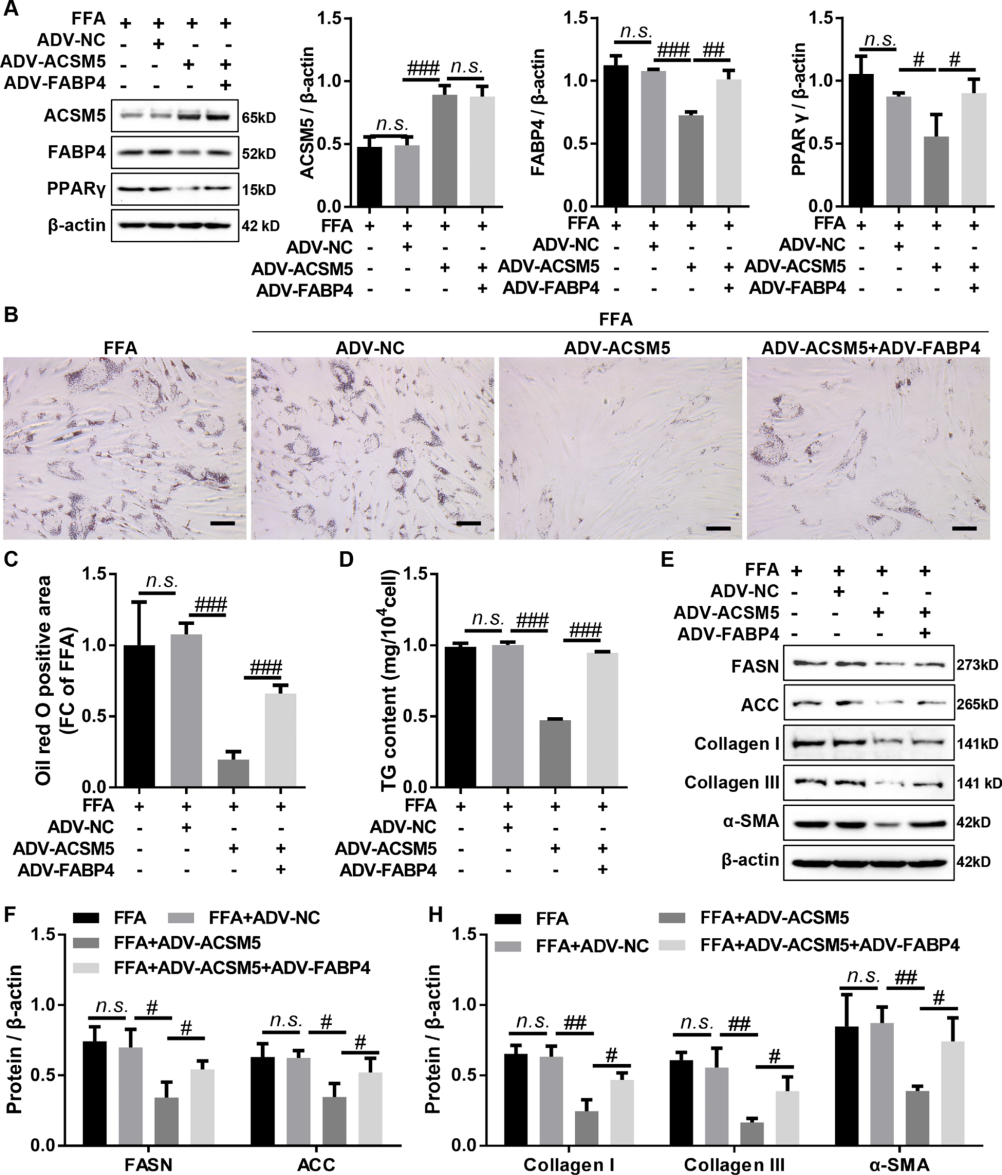
Overexpression of ACSM5 inhibited FFA-induced lipid accumulation and fibrosis in LF cells by regulating the FABP4-mediated PPARγ signaling. (**A**) LF cells infected with ADV-ACSM5 alone or in combination with ADV-FABP4 were treated with FFA for 48 h, and the protein expression of ACSM5, FABP4 and PPARγ in LF cells was detected by western blot. (**B** and **C**) LF cells infected with ADV-ACSM5 alone or in combination with ADV-FABP4 were treated with FFA for 48 h, and oil red O staining was used to detect intracellular lipid accumulation in LF cells. Bar size = 100 nm. (**D**) LF cells infected with ADV-ACSM5 alone or in combination with ADV-FABP4 were treated with FFA for 48 h, and TG assay kit was used to detect TG levels in LF cells. (**E** and **F**) LF cells infected with ADV-ACSM5 alone or in combination with ADV-FABP4 were treated with FFA for 48 h, and the protein expression of FASN and ACC in LF cells was detected by western blot. (**E** and **H**) LF cells infected with ADV-ACSM5 alone or in combination with ADV-FABP4 were treated with FFA for 48 h, and the protein expression of collagen I, collagen III and α-SMA in LF cells was detected by western blot. LF, ligamentum flavum. FFA, free fatty acids. ADV, adenovirus. NC, negative control. ^n.s^.P > 0.05, ^#^P < 0.05, ^##^P < 0.01, and ^###^P < 0.001

To explore whether ACSM5 regulates FFA-induced lipid accumulation and fibrosis in LF cells through inhibiting FABP4-mediated PPAR signaling, we performed a series of rescue experiments in FFA-treated LF cells. The results of western blot disclosed that overexpression of FABP4 significantly attenuated the inhibitory effect of ACSM5 overexpression on the protein expression levels of FABP4 and PPARγ (Fig. 4A), suggesting that ACSM5 inhibited PPARγ signaling pathway activation by downregulating FABP4 in FFA-treated LF cells. Further analysis using oil red O staining and TG assay demonstrated that overexpression of ACSM5 significantly suppressed FFA-induced lipid accumulation and TG content in LF cells, and this effect was significantly reversed by overexpression of FABP4 (Fig. 4B, C and D). Meanwhile, western blot results also demonstrated that overexpression of ACSM5 significantly reduced the protein expression of lipid synthesis genes (FASN and ACC) in LF cells exposed to FFA, and this inhibitory effect was significantly reversed by overexpression of FABP4 (Fig. 4E, F). In addition, our data revealed that overexpression of ACSM5 significantly inhibited the protein expression of fibrosis-related genes (Collagen I, Collagen III, and α-SMA) in LF cells exposed to FFA, which could be reversed by overexpression of FABP4 (Fig. 4E and G). Together, our data demonstrated that ACSM5 played a critical role in regulating lipid accumulation and fibrosis in FFA-induced LF cells by inhibiting FABP4-mediated PPARγ signaling pathway.

### Overexpression of ACSM5 inhibited LF hypertrophy and lipid accumulation by regulating FABP4-mediated PPAR signaling pathway in mice

**Fig. 5:**
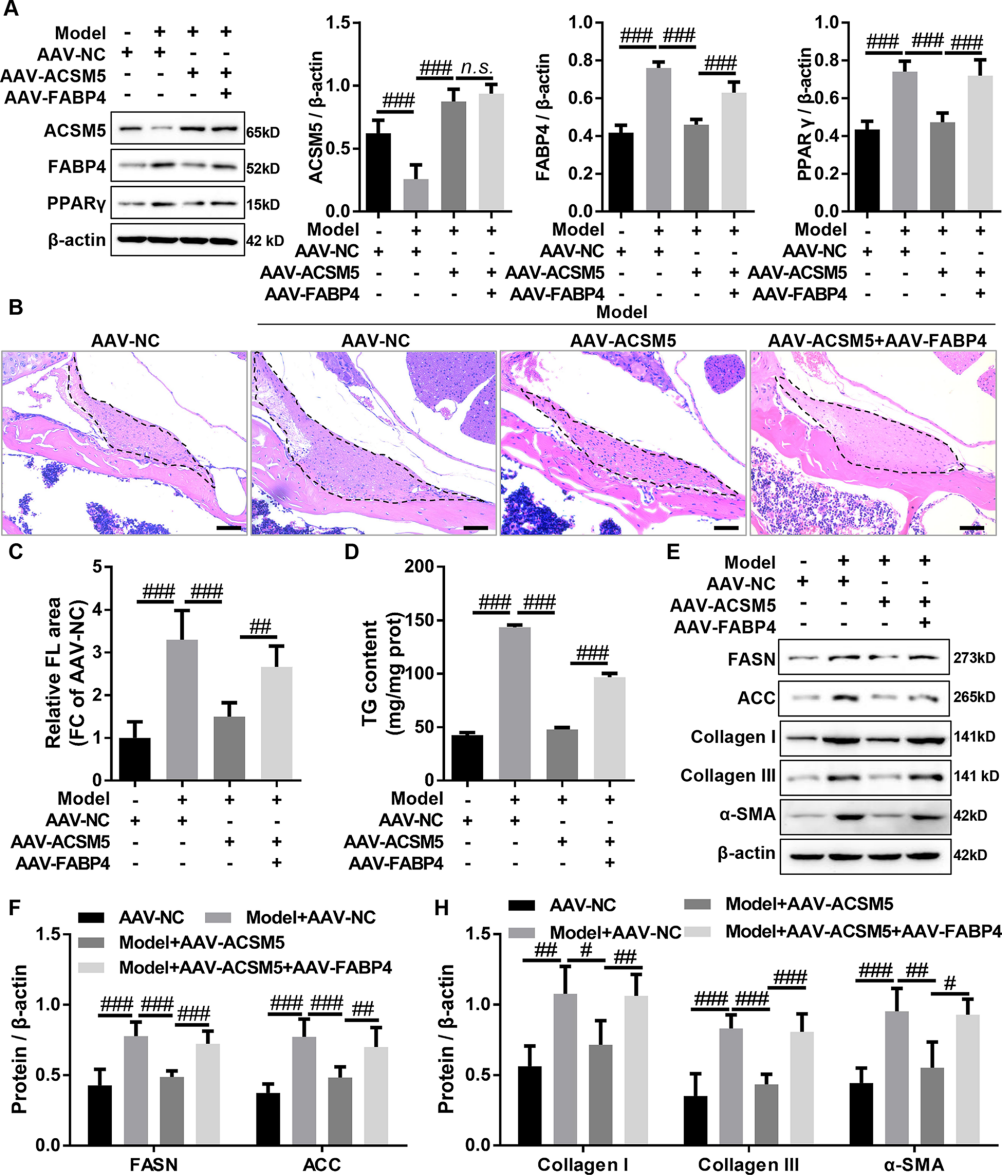
Overexpression of ACSM5 improved LF hypertrophy by inhibiting FABP4-mediated PPAR signaling pathway in mice. (**A**) Western blot was used to detect the protein expression of ACSM5, FABP4, and PPARγ in LF hypertrophy mice treated with AAV-ACSM5 alone or in combination with AAV-FABP4. (**B** and **C**) After ADV-ACSM5 alone or in combination with ADV-FABP4 treatment of mice with LF hypertrophy, the LF area was assessed using HE staining. Bar size = 50 nm. (**D**) After ADV-ACSM5 alone or in combination with ADV-FABP4 treatment of mice with LF hypertrophy, and TG assay kit was used to detect TG levels in LF tissue. (**E** and **F**) After ADV-ACSM5 alone or in combination with ADV-FABP4 treatment of mice with LF hypertrophy, and the protein expression of FASN and ACC in LF tissue was detected by western blot. (**E** and **H**) After ADV-ACSM5 alone or in combination with ADV-FABP4 treatment of mice with LF hypertrophy, and the protein expression of collagen I, collagen III, and α-SMA in LF tissue was detected by western blot. LF, ligamentum flavum. Model, mice with LF hypertrophy. AAV, adeno-associated virus. NC, negative control. ^n.s^.P > 0.05, ^#^P < 0.05, ^##^P < 0.01, and ^###^P < 0.001

To further confirm that ACSM5 delayed LF hypertrophy by inhibiting FABP4-mediated PPARγ signaling pathway, a mouse model of LF hypertrophy, which is highly consistent with human clinical features, was constructed using hydrophobic property. The mice with LF hypertrophy were treated with AAV-ACSM5 alone or in combination with AAV-FABP4, and the expression levels of ACSM5, FABP4, and PPARγ in LF tissues were measured by western blot. The results of western blot indicated that AAV-ACSM5 significantly increased the protein expression levels of ACSM5 as well as decreased the protein expression levels of FABP4 and PPARγ in mice with LF hypertrophy, and this effect could be reversed by co-treatment of AAV-FABP4 and AAV-ACSM5 (Fig. 5A). These results demonstrated that overexpression of ACSM5 inhibited PPARγ signaling pathway by blocking FABP4 in LF hypertrophy tissue. H&E staining results demonstrated that overexpression of ACSM5 significantly inhibited LF hypertrophy in mice, and this inhibitory effect was weakened by overexpression of FABP4 (Fig. 5B, C). Furthermore, the results of TG assay indicated that overexpression of ACSM5 significantly decreased TG levels of LF hypertrophy tissues in mice, and this inhibition was partially reversed by overexpression of FABP4 (Fig. 5D). Meanwhile, western blot results showed that overexpression of ACSM5 significantly inhibited the protein expression of lipid synthesis genes (FASN and ACC) in the LF hypertrophy tissues of mice, and this inhibitory effect could be partially rescued by overexpression of FABP4 (Fig. 5E, F). In addition, the protein expression levels of fibrosis-related genes (Collagen I, Collagen III, and α-SMA) in LF tissues were detected by western blot. The results showed that overexpression of ACSM5 significantly inhibited the protein expression of Collagen I, Collagen III and α-SMA in LF hypertrophy tissues of mice, and this effect was significantly attenuated by overexpression of FABP4 (Fig. 5E, H). Altogether, these outcomes demonstrated that ACSM5 inhibited LF hypertrophy and lipid accumulation by inhibiting the FABP4-mediated PPARγ signaling pathway in mice.

## Discussion

Current study has shown that lipid accumulation is abnormally increased in hypertrophy tissues of the LF [7], but the regulatory mechanism remains unclear. In this study, our study discovered that downregulation of ACSM5 in LF hypertrophy tissues was significantly negatively correlated with lipid accumulation, and overexpression of ACSM5 suppressed LF hypertrophy by inhibiting FABP4-mediated PPARγ signaling pathway to alleviate lipid accumulation.

Our previous in vitro study revealed that DNMT1-mediated ACSM5 hypermethylation resulted in down-regulation of ACSM5 in LF hypertrophy tissues, while blocking DNMT1/ACSM5 axis could promote proliferation and fibrosis as well as inhibit apoptosis in LF cells [13]. Consistent with previous in vitro studies, our in vivo data confirmed that ACSM5 was lowly expressed in LF hypertrophy tissues, and overexpression of ACSM5 could markedly inhibit hypertrophy and fibrosis of LF. Interestingly, our data also showed the lipid accumulation and TG levels were significantly increased in LF hypertrophy tissue and were closely correlated with LF thickness, which consistent with the conclusion of Yamada T et al. [7]. Lipids play important roles in signal transduction, maintaining the integrity of cell membrane structure, and regulating energy metabolism [25]. Abnormal lipid metabolism leads to the accumulation of lipids in cells, resulting in cell dysfunction and death in various tissues such as kidney, brain, heart and skeletal muscle, thereby aggravating the occurrence and development of diseases [26–29]. In this study, our data revealed that overexpression of ACSM5 could significantly inhibit lipid accumulation in LF cells in vitro and in vivo.

With the rapid development of high-throughput sequencing technology, mRNA sequencing has become an effective tool for revealing the pathogenesis of diseases. Based on mRNA sequencing and analysis, we found that ACSM5-regulated DGEs were mainly enriched in pathways related to lipid metabolism and inflammation in LF cells, such as the PPAR signaling pathway. PPARs (PPAR-α, PPAR-β/δ, and PPAR-γ) belong to the ligand-activated nuclear transcription factor superfamily, which regulates energy metabolism, inflammatory response, and lipid homeostasis by binding to ligands and cofactors [30]. Recent studies have shown that the regulatory role of the PPAR signaling pathway in lipid metabolism in several diseases is still controversial [31]. For example, Zhuang et al. demonstrated that amentoflavone inhibited ox-LDL-induced lipid accumulation by suppressing the PPARγ/CD36 signal pathway [32]. Yu et al. reported that upregulation of PPAR signaling, accompanied by increased lipid accumulation, might trigger morphological and structural transitions in longan goldfish eyes [33]. Lee et al. showed that heat-inactivated enterococcus faecalis prevented lipogenesis and high-fat diet-induced obesity by inhibiting lipid accumulation through PPAR-γ inhibition [34]. In this study, in vitro experiments demonstrated that ACSM5 inhibited FFA-induced FABP4/PPARγ signaling pathway activation in LF cells, and further in vivo animal experiments also indicated that overexpression of ACSM5 significantly inhibited the activation of FABP4/PPARγ signaling pathway in LF hypertrophy tissues.

FABPs are a family of intracellular lipid chaperones that coordinate the intracellular lipid response by binding to and redistributing intracellular fatty acids [35]. FABP4 is one of the most characteristic intracellular lipid transporters in the FABPs family, which plays an important regulatory role in lipid metabolism disorders and fibrosis [36–38]. Scifres et al. reported that FABP4 knockdown significantly decreased intracellular lipid accumulation in human trophoblast cells [39]. Song et al. demonstrated that metformin reduced palmitic acid-induced intracellular lipid accumulation in macrophages by inhibiting FABP4 [40]. Chen et al. disclosed that treatment with the FBAP4 inhibitor BMS309403 significantly attenuated lipid deposition and interstitial fibrosis in renal tubular epithelial cells [41]. In this study, we discovered that FBAP4 was significantly upregulated in FFA-induced LF cells and LF hypertrophy tissues, and overexpression of ACSM5 significantly inhibited the mRNA and protein expression of FBAP4 and PPARγ. More and more studies have shown that FABP4 is an upstream gene in the PPAR-γ pathway, which can bind to PPAR-γ and promote its protein stability and nuclear import [22, 42]. Our results revealed that overexpression of ACSM5 inhibited PPARγ signaling pathway activation in FFA-induced LF cells and LF hypertrophy tissue, and the inhibitory effect was significantly reversed by FABP4 overexpression. These data suggested that ACSM5 inhibited PPARγ signaling pathway activation by inhibiting FABP4 in vitro and in vivo. In addition, functional experiments also showed that overexpression of FABP4 significantly abolished the inhibitory effects of ACSM5 on LF lipid accumulation and LF hypertrophy. However, there are still some shortcomings in this study, such as the failure to independently verify the role and mechanism of FABP4 or PPARγ in lipid accumulation and fibrosis of LF, and the failure to explore how ACSM5 inhibits FABP4 expression to protect lipid accumulation and fibrosis of LF, which will be the focus of our subsequent studies.

## Conclusion

In this study, our data revealed that ACSM5 improved LF hypertrophy by inhibiting FABP4-mediated PPARγ signaling pathway to inhibit lipid accumulation. These results suggested that therapeutic strategies directed towards improving lipid deposition may be an effective potential approach to treat LF hypertrophy-induced LSCS.

### Electronic supplementary material

Below is the link to the electronic supplementary material.

Supplementary Material 1

## Data Availability

The datasets used and/or analyzed during the current study are available from the corresponding author on reasonable request.
