# Supplementary material for: ACSM5 inhibits ligamentum flavum hypertrophy by regulating lipid accumulation mediated by FABP4/PPAR signaling pathway
Source: Biol Direct. 2023 Nov 14;18:75. doi: 10.1186/s13062-023-00436-z (PMC10644428; doi:10.1186/s13062-023-00436-z)
Supplement: Supplementary file 1 — Supplementary Material 1 [file 13062_2023_436_MOESM1_ESM.docx]

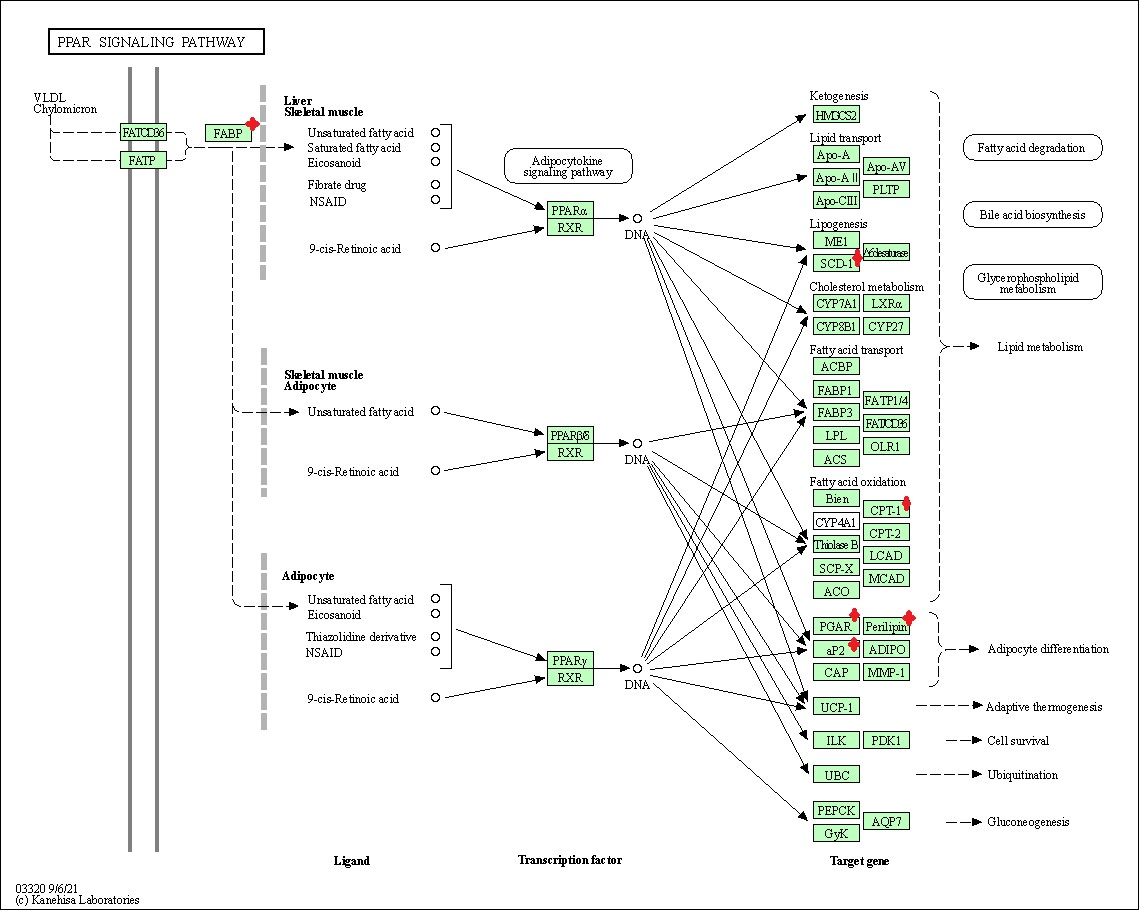


**Figure S1 PPAR signaling pathway.** The red four-pointed stars represent differential genes enriched in this pathway.
